# The New Year: The Association and "The Hospital"

**Published:** 1887-01-01

**Authors:** 


### THE NEW YEAR: THE ASSOCIATION AND "THE HOSPITAL."

Development: A New Application.

The doctrine of development, though no longer possessing the charm of novelty, is still an article of faith and a working hypothesis. It has survived Darwin, and may possibly outlive Mr. Herbert Spencer, who is now by no means young. There is no impropriety in comparing small things with great, still less great things with greater; and if Mr. Spencer constructs a science of sociology out of the analogies which exist between social progress and the development of vegetable and animal organisms, there is no reason why Darwin's ideas should not be borrowed a second time, and used for our guidance in elucidating the history and advancement of the hospital system. Institutions, as well as material organisms, have their embryonic and their more differentiated stage. In the development of the hospital system the mere protoplasmic condition is long since past. We have now arrived at a stage of high differentiation and complexity of structure, with a corresponding degree of specialisation of function.

The Cerebrum: A New Organ.

Some few years ago a new organ was developed in the shape of the *Hospitals Association.* The Association is a rudimentary cerebrum, but though at present rudimentary, it possesses all the potentialities of a highly efficient organ of mind. All that it requires is adequate nutrition, with regular exercise, in order that it may prove itself, like other efficient cerebral systems, of the utmost possible service to the organism of which it is a part. Abundant nutrition is indispensable. The best minds connected with hospitals must become, as many of them have already become, energetic members of the Association. They must see in union and comparison of ideas the possibilities of all kinds of improvement and progress. They must be willing, for the sake of the general good, to lay aside their prejudices and worn-out methods. Seeing that, whether they will or will not, they cannot help being parts of a system, they should gracefully recognise the inevitable, and resolve that they will contribute, to the utmost of their ability, to the general store of wisdom, sympathy, and mutual help.

Work: not Play.

The Association is now at a somewhat critical period of its life. It is feeling the necessity for strong and useful action. The intention of its founders would be altogether frustrated if it should degenerate into a merely ornamental or talking society. There is a vast amount of work—work of the highest importance to hospitals, which imperatively demands to be done. When it is considered how many hundreds, nay, thousands of hospitals there are in Great Britain: how many people are associated with them as subscribers, medical men, matrons, secretaries, nurses, servants, patients—in and out; what vast sums of money, amounting to several millions sterling, are annually spent in their establishment and maintenance,—when all these things are taken into review, it is seen how huge is our hospital system, and how vast and varied is the field of its operations.

A Compendium and a Finger-post.

The Association desires to be a readily available *compendium of hospital knowledge* and a *finger-post* to all classes of wayfarers who may be travelling on hospital highways. That such a compendium would be of the greatest possible value, no one can doubt; that such a finger-post is urgently and constantly needed, no one can deny. We cannot too strongly urge upon all hospital managers, matrons, physicians, surgeons, secretaries, subscribers, and persons who are in any way whatsoever connected with or interested in hospitals, the great advantages which will result to them and their institutions from membership in the Association, and from giving to it the constant help of their individual sympathy and experience.

The Tongue: a Further Development.

A still more recent development of the hospital system is the weekly organ, which in modern times, at any rate, seems to be the necessary accompaniment of any great and growing movement. We refer to THE HOSPITAL. Now that the paper is an accomplished fact, everybody discovers how greatly it was needed, and wonders why it was not started years ago. THE HOSPITAL is to the Association, and through it to the world of charity, what every organisation must have which appeals in so many ways to a mixed public. It is a *tongue.* In these days he who has no tongue, or having one cannot use it, has small chance of making headway in the general scramble. If the hospitals are to maintain their ground and to advance, they must have the means of speaking, when occasion requires, with a *collective voice.* True, each of them can speak separately by circular or other publication of the like kind. But who heeds a circular? A newspaper will be read where a circular would be tossed contemptuously into the fire. THE HOSPITAL does not enter the house like a circular, dressed in the garb and speaking in the tones

A Teacher: not a Beggar.

of a beggar. It goes as a teacher: as a guide and instructor: and it even condescends to amuse. There are those who think that some loss of dignity is incurred when it unbends from the solemnity of economics and statistics and the high mysteries of the medical and surgical arts. Not so! There is a purpose in all it does: a method in its madness. So great an authority in religion and morals as St. Paul avowed his readiness to "become all things to all men" for a sufficient reason. We endeavour to become all things to all men, for the sufficient reason that we wish by every legitimate means to convert all men into supporters and friends of hospitals. That object will not be accomplished by mere logic, or by never so well-arranged bundles of facts and statistics. You might as well throw a brickbat at some men's heads, as attempt to influence them by statistics. Our ambition is to make hospitals live before the eyes and in the minds of men. We wish by variety of articles, by the recital of moving incidents, even by using the arts of the novelist and the story-teller, to make everyone not only see and know, but love the noble work in which hospitals are engaged.

Variety: not Dulness.

We can conceive it possible that a rosy apple might spring out of the ground like a puff-ball, or even adhere to the rock like a limpet. It would still be an apple, and as sweet to the taste as now. But do we not regard the beautiful fruit with all the more pleasure, because the tree from which it grows has a sturdy trunk, spreading branches, whispering leaves, and fragrant blossoms? So with our statistics, facts, and arguments. They will not be less, but more weighty and convincing, because they are accompanied by lighter, more graceful, and more pleasing matter. In any case, we have but one motive in all our work, and that is, to promote by every means in our power the efficiency, economy, and prosperity of hospitals and similar institutions.

Two last Words and "A Happy New Year."

In wishing our readers with all sincerity a "Happy New Year," we desire to say *two* final words. The first is a word of grateful thanks. The Hospital has found readers in numbers beyond our most sanguine anticipations, and in appreciation beyond our utmost deserts. For this our gratitude is deep and sincere. The second is a request. As the cause of hospitals is not ours alone, but that of all good and generous men, we think we may be permitted to ask our readers to do what in them lies to increase our circulation tenfold, being assured that such increase will do more to promote the prosperity and efficiency of those institutions than any other means that are now available. In the hope that the coming year may be the brightest and best in the history both of the hospitals and their supporters, we conclude by once more wishing each and all "A Happy New Year."

